# Influence of pulsed electromagnetic field (PEMF) therapy on osteoarthritis in dogs

**DOI:** 10.1186/s12917-025-05036-9

**Published:** 2025-10-03

**Authors:** S. Šutalo, L. Klasen, A. Tichy, O. Harms

**Affiliations:** 1 Small Animal Clinic, Freie Universität Berlin, Berlin, Germany; 2 Animal Hospital Germersheim, Germersheim, Germany; 3 Platform Bioinformatics and Biostatistics, University of Veterinary Medicine, Vienna, Austria

**Keywords:** Osteoarthritis, Degenerative joint disease, Canine, Lameness, Pulsed electromagnetic field

## Abstract

**Objective:**

This randomized, double-blind, placebo-controlled clinical trial investigated the therapeutic efficacy of pulsed electromagnetic field (PEMF) therapy in dogs diagnosed with chronic osteoarthritis (OA).

**Methods:**

Twenty-one dogs with radiographically confirmed OA in at least one limb were randomized into treatment (*n* = 10) and placebo (*n* = 11) groups. PEMF therapy was administered over six weeks. Gait symmetry indices for peak vertical force and vertical impulse were assessed using kinetic analysis at baseline (Day 0), mid-treatment (Day 21), and post-treatment (Day 42). Lameness and pain scores, additional treatments, and dropout rates were recorded. Owners completed the Liverpool Osteoarthritis in Dogs (LOAD) questionnaire at each time point. An Overall Treatment Effectiveness (OTE) score was computed based on objective and subjective outcomes.

**Results:**

PEMF-treated dogs exhibited significant improvements in gait symmetry by Day 42 (*p* = 0.030). LOAD scores declined steadily in the treatment group, although no statistically significant differences were observed between groups. This trend may suggest a potential reduction in pain and improvement in mobility. No significant between-group differences were found for the OTE score.

**Conclusion:**

PEMF therapy appears to be a safe, non-invasive, and potentially effective adjunctive or stand-alone modality for the management of pain and mobility impairment associated with osteoarthritis in dogs. Further research is warranted to confirm long-term efficacy and optimize treatment protocols.

## Introduction

Osteoarthritis (OA) is a prevalent degenerative joint disorder in dogs, marked by progressive cartilage degradation, chronic pain, and reduced mobility. It substantially affects the quality of life of both affected dogs and their owners [1, 2]. Medium to large breeds are particularly predisposed, and up to 20% of the adult canine population may be affected [3, 4]. As OA is irreversible, therapeutic strategies primarily aim at symptom control and functional improvement through multimodal interventions [5–7].

Among emerging non-pharmacological approaches, pulsed electromagnetic field (PEMF) therapy has gained increasing attention [8]. Although PEMF has been utilized in human and veterinary medicine for decades, its mechanisms and clinical value remain partially understood [9].

PEMF therapy is based on the application of low-frequency electromagnetic fields, leveraging Faraday’s law of induction and ion cyclotron resonance to modulate ion channel activity and intracellular signaling [10, 11]. These electromagnetic fields can penetrate tissues without inducing heat and influence cellular functions, including gene expression [12–14]. Reported physiological effects include enhanced microcirculation, increased erythrocyte membrane potential, improved tissue oxygenation, and reduction in nitric oxide levels and mitochondrial free radical production [15]. Additionally, PEMF has beneficial effects on angiogenesis and lymphatic flow, which help reduce pain and edema while enhancing bone and tissue healing [16–18].

Experimental studies have shown that PEMF positively influences chondrocyte metabolism, enhancing glycosaminoglycan and protein synthesis, and exerting chondroprotective effects, particularly in early stages of OA [19–21]. In rodent models, PEMF has been shown to reduce cartilage degeneration and support subchondral bone integrity [22, 23].

Clinical studies in humans have reported functional improvements and pain relief following PEMF therapy. However, meta-analyses suggest that PEMF’s efficacy may be comparable to other conservative treatments [24–28]. In canine studies, PEMF has demonstrated promising effects in improving mobility and reducing pain, although findings remain inconsistent. Notably, variation in treatment protocols—frequency, duration, and target site—complicates comparisons across studies [8, 29–33].

The present study aimed to evaluate the therapeutic efficacy of a six-week PEMF treatment protocol in dogs with chronic OA, using both objective gait analysis and subjective owner-reported outcomes.

## Materials and methods

### Study design

A randomized, double-blind, placebo-controlled clinical trial was conducted at the Animal Hospital Germersheim (TiGe) between May 2024 and April 2025. Twenty-one client-owned adult dogs of various breeds and ages, each diagnosed with chronic osteoarthritis (OA), were enrolled after meeting predefined inclusion and exclusion criteria.

All dogs underwent comprehensive physical, neurological, and orthopedic examinations, including radiographic assessment to confirm OA. Lameness was graded using a six-point scale (0 = no lameness to 5 = non-weight-bearing), and joint pain on palpation was scored on a five-point scale (0 = no pain to 4 = palpation not tolerated) [34]. Measurements were recorded at baseline (Day 0), midpoint (Day 21), and end of study (Day 42).

#### Inclusion criteria:

- Dogs older than 12 months with radiographically confirmed OA in one or more joints.
- Evidence of mobility restriction.
- Dogs on stable, long-term OA medication regimens (unchanged for at least 4 weeks) were eligible.

#### Exclusion criteria:

- OA-related surgery within the past six months or during study.
- Multimorbidity likely to affect results.
- Pregnancy.
- Dogs receiving recent or newly initiated NSAID, corticosteroid, or opioid therapy within 4 weeks of Day 0 were excluded.
- Missing more than 30% of treatments.

### Randomization

Dogs were randomly assigned to the treatment or placebo group using a computer-generated randomization list. Allocation was implemented through color-coded therapy mats (orange or black), which were visually identical but functionally distinct. The assignment of which color corresponded to the verum or placebo device was determined by coin flip prior to study start and documented in a sealed blinding form.

Blinding was ensured for both investigators and dog owners throughout the study. Only the clinical supply manager, who was responsible for device preparation, was unblinded. The sealed blinding record remained inaccessible until study completion.

### PEMF therapy

The treatment was administered using BEMER® devices delivering low-intensity pulsed electromagnetic fields (100–150 µT). Dogs in the treatment group received two daily 8-min sessions over a period of six Weeks. Treatment intensity increased weekly: 22 µT in Week 1, 44 µT in Week 2, and 60 µT from Week 3 to Week 6. The placebo group used visually identical mats lacking active electromagnetic output. Frequency alternated between 30 and 10 Hz with 3-s pauses.

### Gait analysis

Objective gait analysis was performed using a pressure-sensitive walkway system (Zebris FDM, 2 × 2 m, 200 Hz, Zebris Medical GmbH, Germany), which records ground reaction forces in three orthogonal directions: mediolateral (Fx), craniocaudal (Fy), and vertical (Fz). Gait parameters were recorded at both walk and trot.

Each dog underwent an acclimatization period to become familiar with the testing environment. During data collection, dogs were walked in a straight line across the pressure plate by their owners, using a loose leash and minimal physical or verbal influence to ensure a natural and undisturbed gait pattern.

To control for velocity-dependent effects on kinetic parameters, gait speed was continuously monitored using motion sensors integrated into the Zebris system. Trials were accepted only if velocity remained within predefined ranges: 0.5–1.2 m/s for walk and 1.5–2.2 m/s for trot.

Only trials meeting strict quality criteria—correct gait type, accurate paw placement without oversteps or misstrikes, and velocity within the defined range—were included in the analysis. For each dog, five valid gait cycles were recorded at both gait types.

Vertical force parameters (peak vertical force [F], mean vertical force [F_M_], and impulse [I]) were normalized to body weight. A symmetry index (SI) was computed for F and I using the formula:

$$\mathrm{SI}\;\left(\%\right)\;=100-\left[\left(\mathrm{Fa}/\mathrm{Fc}\right)\times100\right]$$

where Fa and Fc denote values from the affected and contralateral limbs, respectively [35]. An SI > 6% was classified as indicative of lameness, while perfect symmetry is represented by an SI of 0%. Optimal cutoff values for differentiating between lame and non-lame dogs based on SI change are approximately 3.5% for I and 3.7% for F. Changes in SI over time were analyzed for inter- and intra-group comparisons.

### Overall Treatment Effectiveness (OTE)

The OTE score combined multiple outcome measures, including SI metrics, clinical assessments of lameness and pain, long-term medication use, study dropouts, and the need for additional analgesics or physiotherapy [36]. Scores ranged from −1 (worsened) to + 1 (improved) [37].

### LOAD questionnaire

Owners completed the Liverpool Osteoarthritis in Dogs (LOAD) questionnaire at each timepoint [38]. Scores ranged from 0 (no symptoms) to 52 (extreme OA). Two additional questions rated general health and quality of life (1 = very poor, 10 = excellent).

### Statistical analysis

All statistical analyses were conducted using IBM SPSS Statistics (Version 29). SI values for F and I were analyzed for inter- and intra-group differences at the timepoints Day 0, Day 21, and Day 42 using non-parametric methods.

A Type III fixed effects model was used to assess the effects of treatment, timepoint, and gait type on the OTE score as well as on SI values for F and I. Pairwise comparisons were conducted to evaluate group differences in SI across timepoints.

Additionally, statistical analyses were performed using a linear mixed model with fixed effects for treatment group, timepoint, gait type, and their interactions. Dogs were modeled as random effects to account for repeated measures within subjects. Post hoc pairwise comparisons were performed for all group × time × gait combinations. The Sidak correction was applied where appropriate to adjust for multiple testing.

One-sample t-tests against zero were performed within each group, timepoint, and gait type to determine whether SI values significantly deviated from perfect symmetry (SI = 0).

The Mann–Whitney U test was applied to compare LOAD scores and OTE between the treatment and placebo groups. Within-group changes from baseline (Day 0) to Day 21 and Day 42 were assessed using the Wilcoxon Signed-Rank Test.

A *p*-value < 0.05 was considered statistically significant. Normally distributed data are presented as mean ± standard deviation (SD).

## Results

Twenty-one dogs were enrolled, with 11 assigned to the placebo group and 10 to the PEMF treatment group. One placebo group dog died early in the study due to unrelated causes. One dog in the treatment group was euthanized between Days 21 and 42 following the onset of progressive pelvic limb ataxia, which was unrelated to the initial diagnosis of forelimb osteoarthritis. Although the dog met all inclusion criteria at baseline, degenerative myelopathy was suspected later during the study. Data from both dogs were excluded from all subsequent analyses, and only those subjects who completed the study protocol were included in the statistical evaluation.

The dogs ranged from 2.6 to 14.5 years old (TG: 9.01 ± 3.1 years; PG: 8.44 ± 3.2 years) and weighed between 9 and 42 kg (TG: 29.7 ± 9.1 kg; PG: 29.2 ± 8.2 kg). The population included a variety of breeds, with both forelimb and hindlimb lameness observed. In the treatment group, 40% of dogs had forelimb, 50% hindlimb, and 10% both. In the placebo group, 20% had forelimb, 60% hindlimb, and 20% both. Five dogs in each group received long-term medications; additional NSAID or supplement usage was noted but consistent between groups (Appendix Table 1).

The mean F values at Day 0 for walk and trot were 137.66 ± 43.63% and 196.86 ± 67.21% in the placebo group (PG), and 154.07 ± 63.72% and 211.16 ± 88.98% in the treatment group (TG). At Day 21, F values in walk and trot were 139.61 ± 43.99% and 204.89 ± 75.84% in PG, and 158.33 ± 68.67% and 226.49 ± 98.49% in TG. By Day 42, F in walk and trot measured 136.05 ± 41.70% and 206.31 ± 58.19% in PG, and 150.38 ± 63.66% and 229.85 ± 99.18% in TG.

The mean I values at Day 0 for walk and trot were 53.32 ± 21.76% and 39.45 ± 14.12% in PG, and 60.24 ± 25.57% and 41.07 ± 18.44% in TG. At Day 21, I values in walk and trot were 52.76 ± 19.99% and 37.30 ± 13.63% in PG, and 61.99 ± 31.32% and 38.82 ± 17.95% in TG. By Day 42, I values in walk and trot were 53.97 ± 22.77% and 38.04 ± 11.36% in PG, and 52.26 ± 23.70% and 41.51 ± 20.43% in TG.

The mean SI F values at Day 0 for walk and trot were 7.76 ± 11.6% and 10.27 ± 17.7% in PG, and 3.52 ± 5.8% and 2.15 ± 16.5% in TG. At Day 21, SI F values in walk and trot were 4.14 ± 12.6% and 13.94 ± 17.6% in PG, and 3.63 ± 4.6% and 1.28 ± 17.2% in TG. By Day 42, SI F in walk and trot measured 9.40 ± 12.5% and 14.95 ± 20.9% in PG, and 0.85 ± 6.3% and –0.43 ± 18.4% in TG.

**Fig. 1:**
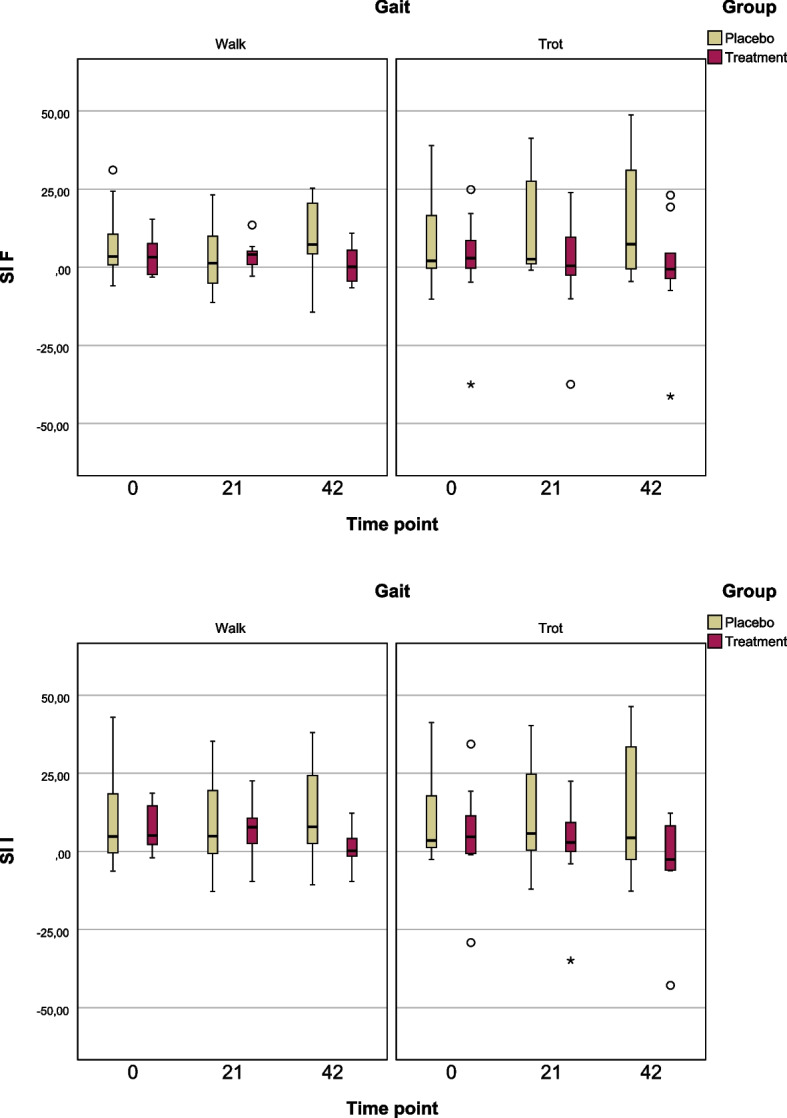
Boxplots illustrating the distribution of symmetry indices (SI F and SI I) across three time points (Day 0, Day 21, and Day 42) during both walk and trot gaits in the placebo and PEMF treatment groups The data suggest that PEMF treatment may contribute to improved gait symmetry and reduced variability over time, particularly in comparison to the placebo group, where greater asymmetry and dispersion are evident at later time points

The mean SI I values at Day 0 for walk and trot were 10.36 ± 15.3% and 12.04 ± 16.3% in PG, and 7.74 ± 7.1% and 5.13 ± 16.2% in TG. At Day 21, SI I values in walk and trot were 8.62 ± 14.8% and 11.52 ± 18.1% in PG, and 7.44 ± 8.9% and 2.87 ± 15.8% in TG. By Day 42, SI I values in walk and trot were 12.67 ± 17.1% and 12.69 ± 22.3% in PG, and 1.30 ± 6.3% and –3.62 ± 16.3% in TG (Fig. 1).

### Gait symmetry indices

The symmetry index of peak vertical force (SI F) showed a significant main effect for the treatment group (*p* = 0.003). Pairwise comparisons revealed no differences at baseline or Day 21, but a statistically significant improvement was observed by Day 42 (*p* = 0.030), indicating a delayed treatment effect. Symmetry index values in the treatment group approached zero by the end of the study, suggesting restored limb symmetry.

**Fig. 2:**
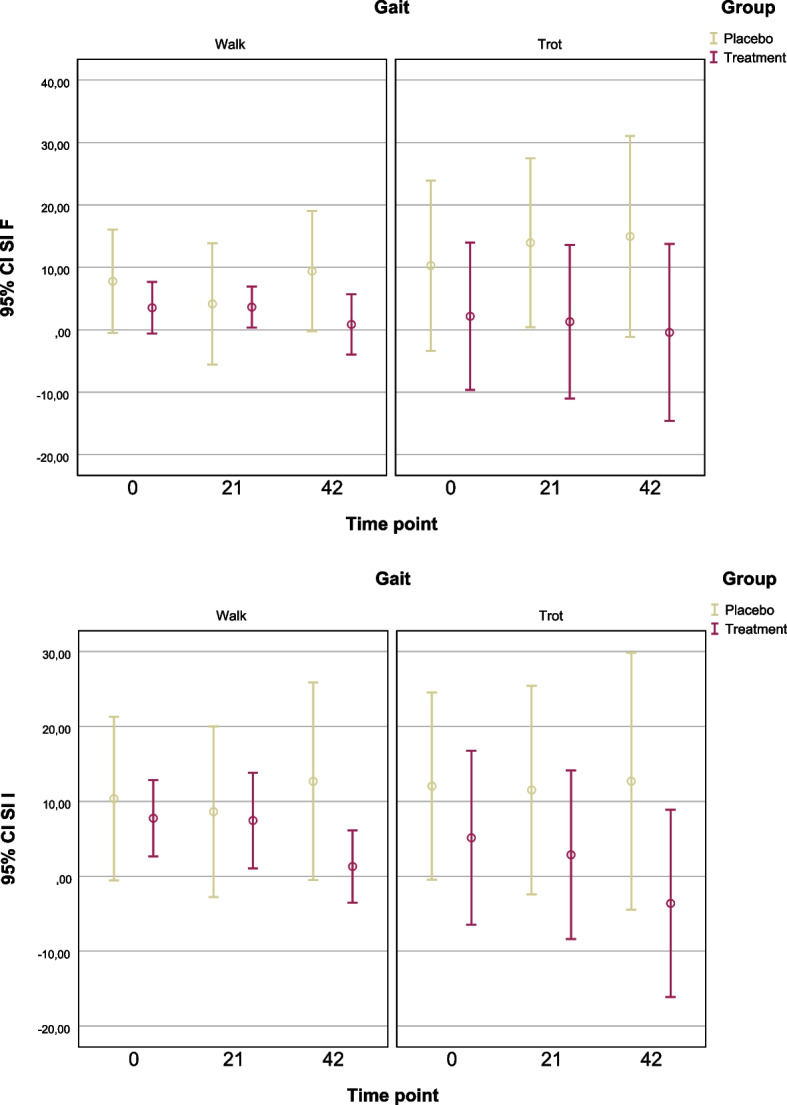
Mean symmetry indices with 95% confidence intervals for peak vertical force (SI F) and vertical impulse (SI I) at Days 0, 21, and 42, shown separately for walk and trot gaits in the placebo and treatment groups In the treatment group, both SI F and SI I values generally decreased over time, particularly at Day 42, suggesting improved gait symmetry. In contrast, the placebo group exhibited higher variability and less consistent improvement across both parameters and gaits

Complementary one-sample t-tests showed that treatment group SI F values no longer differed significantly from zero by Day 42, further indicating normalization of gait. The placebo group, in contrast, maintained elevated and variable SI F values (Fig. 2).

### LOAD questionnaire

**Fig. 3:**
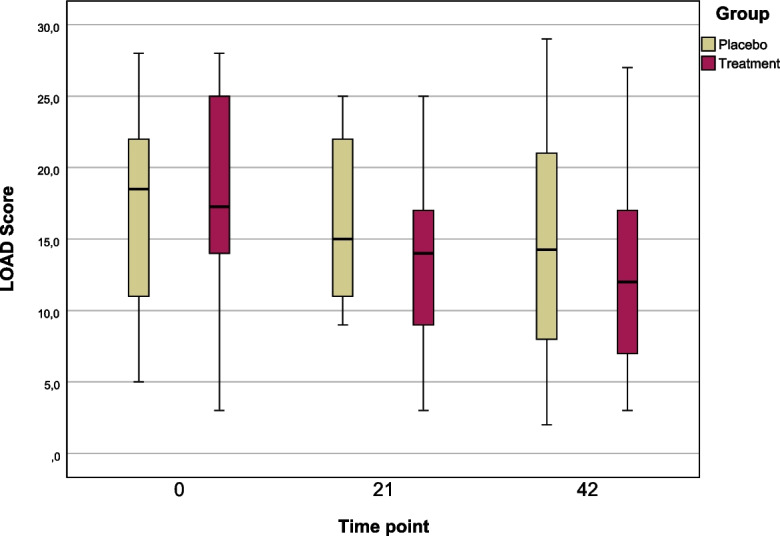
Boxplot visualization of Liverpool Osteoarthritis in Dogs (LOAD) scores at Days 0, 21, and 42 for the placebo and treatment groups

LOAD scores decreased over time in the treatment group; however, no statistically significant differences were observed between groups at any timepoint. The placebo group showed no meaningful change. Additional quality of life and general health scores remained consistently high across both groups (Fig. 3).

### Overall Treatment Effectiveness (OTE)

No significant between-group differences in OTE scores were observed. Median scores remained at zero in most conditions. Greater variability was noted in the placebo group at trot on Day 42, but without statistical significance (all *p* > 0.05).

## Discussion

This study suggests that PEMF therapy may contribute to improved gait symmetry in dogs with OA, with statistically significant improvement shown only at Day 42. These results support the potential of PEMF as a delayed-acting therapeutic intervention for canine osteoarthritis (OA).

The delayed response aligns with prior studies indicating PEMF effects may peak several weeks after initiation [39, 40]. Variability in treatment duration, frequency, and intensity across studies may influence therapeutic outcomes. In human medicine, parameters such as 200 Hz with a continuous pulse for 15 min are commonly used to downregulate pain pathways and induce analgesia [41]. In contrast, lower frequencies, such as 50 Hz, may promote vasodilation, potentially alleviating chronic periarticular swelling and influencing pain and lameness via different mechanisms [42, 43]. In the present study, treatment sessions lasted 8 min and employed a cyclic pulse frequency alternating between 30 and 10 Hz. These relatively short sessions, combined with low field intensities and cycling frequencies, may have limited the observed therapeutic effect. Future research should aim to identify optimized treatment parameters tailored to specific clinical objectives and pathophysiological targets.

Although objective gait analysis is a validated method for detecting lameness, its ability to capture joint-specific changes is limited [44–46]. Furthermore, dogs with mild or multifocal OA may show less measurable improvement due to intrinsic variability [47, 48]. Our use of symmetry indices aimed to normalize for inter-individual differences, but high variance still reduced statistical power.

Pooling data across different joints and limbs may have obscured joint-specific treatment effects. Comparable issues in human OA research suggest joint heterogeneity may mask therapy responses [49–51]. Subgroup analysis based on limb or joint location could improve sensitivity in future studies. Additionally, older and more severely affected dogs may respond less robustly than younger, less advanced cases.

Although the LOAD scores in the treatment group showed a downward trend over the course of the study, no statistically significant between-group differences were detected. It is possible that this reflects a combination of small sample size, high inter-individual variability, and limitations in sensitivity of the LOAD questionnaire under certain conditions. Notably, the additional owner-reported scores for general health and quality of life remained consistently high across both groups throughout the study. This may indicate a ceiling effect, in which high baseline ratings limit the ability to detect meaningful subjective improvements over time. Such effects have been reported in other clinical trials using owner-based scoring systems, particularly in well-managed or mildly affected patient populations [52–54].

The Overall Treatment Effectiveness (OTE) score yielded less conclusive results, potentially due to its integration of heterogeneous variables. Dogs on concurrent medication may have appeared clinically stable or improved due to analgesia, without reflecting PEMF-specific effects. This confounding influence limits the usefulness of the OTE as a stand-alone measure. Subjective and objective outcomes may also diverge, particularly when treatment effects are modest or multifactorial.

Limitations of this study include a small sample size, population heterogeneity, and possible underdosing. Despite these limitations, findings indicate a measurable benefit of PEMF in improving gait symmetry and reducing OA-associated symptoms in dogs. Further studies should evaluate the long-term efficacy, refine treatment parameters, and consider stratified analyses by joint and disease severity.

## Conclusion

This study provides evidence that pulsed electromagnetic field (PEMF) therapy is a non-invasive, well-tolerated, and potentially effective treatment for improving gait symmetry and reducing clinical symptoms associated with osteoarthritis in dogs. While significant improvements were observed only after several weeks of therapy, these findings suggest that PEMF may serve as a valuable adjunctive or stand-alone intervention in the long-term management of canine OA.

To substantiate and expand upon these results, further research is recommended. Future studies should include larger and more homogeneous samples, extended observation periods, and standardized PEMF parameters to determine optimal therapeutic protocols. Joint-specific analyses may also clarify which anatomical sites and OA severities respond most effectively to treatment.

## Figures and Tables

**Table 1:**
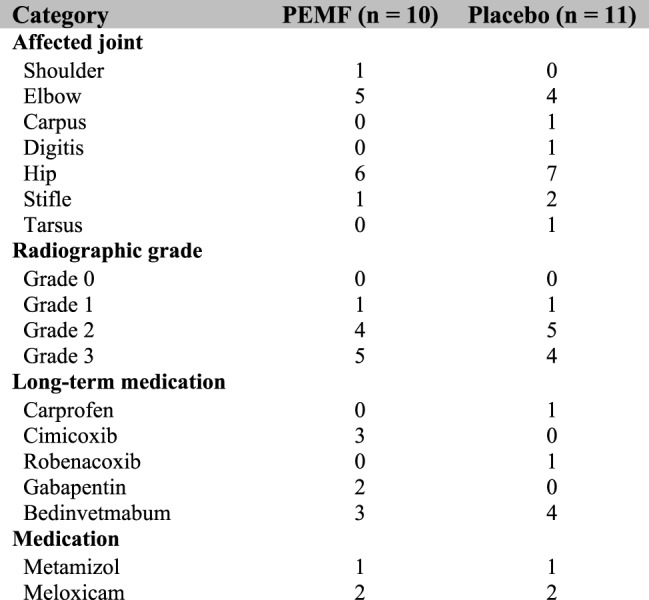
Demographic data and medication

## Data Availability

All data generated or analyzed during this study are available from the corresponding author upon reasonable request.
